# Advances in Polyhydroxyalkanoate (PHA) Production, Volume 4

**DOI:** 10.3390/bioengineering13030269

**Published:** 2026-02-26

**Authors:** Martin Koller

**Affiliations:** Institute of Chemistry, University of Graz, NAWI Graz, Heinrichstrasse 28/IV, 8010 Graz, Austria; martin.koller@uni-graz.at; Tel.: +43-316-380-5463

## 1. Introduction

### 1.1. What Drives the Currently Increasing Interest in Polyhydroxyalkanoates?

Over the last few decades, polyhydroxyalkanoate (PHA) biopolyesters, produced by a vast variety of natural and genetically engineered microorganisms, mainly from the kingdoms of bacteria and archaea, have gained increasing attention in the scientific community and in various industries. PHAs are primarily recognized as a sustainable, biodegradable, and biocompatible alternative to conventional petrochemical plastics, which is crucial for addressing the prevailing global plastic pollution crisis [1,2]. The key reasons for their rising importance include, but are not limited to, the following:

- Susceptibility towards biodegradation: Unlike most traditional plastics that persist in the environment for hundreds of years, PHAs can fully biodegrade into water, carbon dioxide, biomass, and, in anaerobic settings, methane through the action of common microbial species found in various natural environments, such as soil, freshwater, and marine water, without leaving behind persistent microplastics [3,4,5].
- Tapping renewable resources: PHAs are produced biologically through the bioconversion of renewable carbon sources, such as carbohydrates, plant- and animal-derived lipids, alcohols such as glycerol, and, increasingly, second-generation feedstocks such as carbonaceous waste and agro-industrial residues (e.g., rice or wheat straw, whey, molasses, paper waste). Upcycling such waste streams into fermentation feedstocks for PHA biosynthesis aligns excellently with the nowadays highly topical principles of the “circular bioeconomy” and “circular bioengineering”. This strategy contributes to reducing reliance on fossil fuels and managing waste in a sustainable manner. Moreover, it helps fulfilling several of the Sustainable Development Goals defined by the United Nations (UN SDGs) and the currently pursued environmental goals to fight climate change, such as those described in the European Green Deal [6,7,8,9].
- Reducing plastic pollution: The use of PHAs helps mitigate the accumulation of plastic waste in landfills and oceans, which is a significant environmental and health concern, and avoids formation of nano- and microplastic particles, with harmful effects that we are only just beginning to assess and to fully understand [10,11].
- PHAs feature versatile properties and tunable characteristics: Tailored polymers are accessible. The ability to control the monomer composition and structure (e.g., short-chain length (*scl*-PHA) vs. medium-chain length (*mcl*-PHA), or copolymers and terpolymers) enables the fine-tuning of material properties, from rigid thermoplastics to flexible elastomers, to suit specific applications. To date, over 150 different hydroxy acids, acting as PHA monomers, have been identified. This allows for the creation of a wide “PHAmily” of homo- and heteropolyesters with a remarkably broad range of physical and mechanical properties [12]. These properties range from rigid, crystalline, and brittle (such as polypropylene and other established thermoplastics) to flexible and elastic (such as rubber-like elastomers), and can be tailored for specific applications. In this context, novel microbial production strains are currently explored, which feature unique substrate spectra and versatile composition, being made up of unprecedented building blocks, such as species belonging to the emerging *Aneurinibacillus* genus [13]. Apart from the monomeric composition, diverse PHAs differ in intramolecular structure (random or blocky-structured distribution of monomers within polymer chains) and molecular mass and polydispersity. This diversity gave rise to the introduction of the terminus “PHAome” in the scientific literature by leading researchers in this field [14].
- To process PHAs, there is no need to reinvent the wheel: PHAs can be processed using existing conventional and emerging plastic manufacturing equipment (e.g., injection molding, melt extrusion, blow molding, film extrusion, compression molding, additive manufacturing (3D printing), melt spinning, electrospinning), thus facilitating their integration into current industrial production processes [15,16,17].
- PHAs are non-toxic and biocompatible: They break down into natural metabolites occurring in the human metabolism such as the ketone body 3-hydroxybutyrate, the most frequently occurring PHA building block, which is a normal constituent of human blood [18,19,20].

The discussed properties make PHAs ideal for expanding towards high-end applications, such as in the biomedical field, including biodegradable sutures and meshes, tissue for the treatment of inguinal hernia, bone graft substitutes, nerve conduits, cardiovascular patches, and systems for controlled drug delivery. In this context, poly(4-hydroxybutyrate) (P(4HB))-based products have already received FDA approval for clinical use [21]. To an increasing extent, companies are replacing fossil plastics in the production of single-use materials (cups, drinking straws, packaging, containers, etc.) with PHAs; here, the advent of the easily processible and versatile copolyester poly(3-hydroxybutyrate-*co*-3-hydroxyhexanoate) (P(3HB-*co*-3HHx)—originally described and patented by Isao Noda and now produced at large scale by various companies, especially in Asia and the US—has strongly contributed to replacing conventional plastics in low-end applications [22,23,24]. Other regulatory and market drivers currently boosting the interest in PHAs are the growing public awareness of plastic pollution and supportive government regulations, such as those within the European Green Deal [25] and EU’s Single-Use Plastics (SUP) Directive [26]; they are driving increased demand for sustainable materials and pushing companies toward PHA solutions. Hence, it is both an increasing awareness of public and regulatory bodies and the customer as the ultimate decision maker on the market, who increasingly make PHAs the “greenest plastic so far” [20].

### 1.2. The Current Key Trends in PHA Research and Manufacturing

Despite challenges, such as high production costs compared to petroleum-based plastics, ongoing research into using inexpensive waste feedstocks and more efficient production methods; hence, advanced bioengineering is continually improving the commercial feasibility of PHAs. For this reason, several major directions are currently being followed, focusing on reducing the high production costs and expanding top-notch applications, driven by a strong push for sustainable alternatives to conventional fossil-based plastics. Key recent research trends include the following:

- Sustainable and inexpensive production by waste valorization: A key trend is using low-cost, renewable waste streams as feedstocks for microbial fermentation, such as agricultural residues (e.g., grape pomace, corn stover, rice bagasse) [27,28], food waste [29,30,31], waste cooking oil [32,33,34], dairy industry waste (whey) [35,36], industrial exhaust gases [7,37,38], methane [39,40], syngas [41,42], and sludge from wastewater treatment plants [43,44]. This integrates PHA production into the aspired circular bioeconomy and keeps environmental impact to a minimum [45,46].
- Engineering microbial strains and novel synthetic biology tools: To a steadily increasing extent, PHA-related research resorts to advanced synthetic biology techniques such as metabolic pathway engineering and emerging gene-editing and recombinant gene technology approaches such as “gene scissors” (e.g., CRISPR/Cas9 systems) to develop powerful production strains (e.g., *Cupriavidus necator*, *Pseudomonas putida*, halophilic bacteria and haloarchaea, natural non-producers such as *Escherichia coli*, and even plant systems) that can efficiently convert diverse substrates into PHA biopolyesters of tailored composition [47,48,49]. Precise control over the expression levels of PHA synthase and other key enzymes of PHA pathways can optimize production rates [50,51], and increased cultivation performance under low aeration can be achieved via promoter engineering [52,53]. More recently, cell morphology engineering has emerged, maximizing the cultivation and downstream processing efficiency of PHA-accumulating bacteria [54,55,56].
- Advanced bioprocesses for optimized bioengineering: Development of novel fermentation strategies—such as mixed microbial cultures (MMCs) in non-sterile conditions (using “feast–famine” feeding regimes) [57,58], continuous cultivation systems [59], cell retention/recycling systems to covert highly diluted substrate streams [60,61], photobioreactor systems for light-driven PHA production by phototrophs [62,63], and sophisticated gas fermentation systems to convert CO_2_ [64], syngas [42], or methane [65] into PHA-rich biomass—constitute the most striking trends in novel PHA bioengineering.
- Cost-effective downstream processing for PHA recovery from microbial biomass: Innovation in PHA recovery methods is crucial in order to make the entire PHA manufacturing process sustainable. Here, focus is being dedicated to the development of environmentally benign techniques such as using “green”, halogen-free solvents, enzymatic digestion of non-PHA cell mass by hydrolases cocktails, use of ionic liquids, supercritical solvents, switchable ionic surfactants, disrupture of hyperosmotic cells in hypotonic media, programmed cell autolysis, mechanical cell disruption methods (e.g., high pressure homogenization, ultrasonication, bead mills), and even biological methods such as extraction of unscratched PHA granules by insect larvae after digestion of non-PHA biomass. All these techniques are being developed to replace traditional hazardous and energy-intensive PHA recovery processes, typically resorting to halogenated solvents [66,67,68].
- Advanced material properties and modifications are being attained by designing PHA-based polymer blends and composites: To overcome inherent limitations of certain PHAs (e.g., brittleness of poly(3-hydroxybutyrate) (P(3HB)), low thermal stability, suboptimal crystallization rate), blending with other polymers (such as polylactic acid (PLA), poly(ε-caprolactone) (PCL), cellulose fibers or nano-whiskers, lignin, lignocellulose such as bagasse), and inorganic nanofillers (carbon nanotubes, hydroxyapatite, nano-clays) is a key research area. This creates new materials with tailored mechanical, thermal, and barrier properties [69,70,71]. Moreover, specific plasticizers such as glycerol, polyethylene glycol, tributyl citrate, sorbitol, or lauric acid, are frequently applied by manufactures to increase processability of PHAs [72,73]. Ingredients such as citric acid can be integrated into the PHA bulk to make the material functional, e.g., antibiotic or antioxidative, to be used as active food packaging [74].
- As overarching trend, “Next Generation Industrial Biotechnology” (NGIB) is currently considered one of the most promising paths towards efficient production of tailor-made PHA biopolyesters. This trend comprises synthetic biology tools to fine-tune halophilic production strains such as *Halomonas* sp., the use of inexpensive substrates and cultivation media (sea water), cheap and open bioreactor systems operated in continuous mode, and facile PHA recovery from biomass [75,76].

To summarize, currently ongoing research in PHA biopolyesters is increasingly interdisciplinary, leveraging materials science, microbiology, synthetic biology, biotechnology, bioinformatics, and bioengineering to make PHAs both an economically viable and sustainable alternative to conventional plastics. Focusing on the bioengineering aspects of PHAs, the fourth Special Issue dedicated to advances in this field was launched by *Bioengineering*. As the Editor of the journal’s *Biochemical Engineering* section, I feel honored to have been invited to play a guiding role in this Special Issue. Again, different aspects of current research are mirrored in this edition, contributed by emerging and established scientists based in a total of eight countries across three continents. It is especially important to emphasize that time has been dedicated to the emerging trend of using gaseous substrate streams to generate PHA homo- and heteropolyesters by recombinant organisms, use of alternative inexpensive carbon sources, and application of extremophilic bacterial and haloarchaeal production strains for energy-saving and easily scalable PHA production from heterotrophic feedstocks. Moreover, novel tools for quantification of the monomeric composition of PHAs are presented. The subsequent paragraphs highlight the eight individual contributions.

## 2. Individual Contributions

An overarching review article contributed by Anindya Mukherjee from Go!PHA, an international organization actively advocating for the formulation of supportive policies and targeted investments to accelerate the development and commercialization of bio-based, biodegradable, and compostable materials such as PHAs, and the Editor of this Special Issue details the aspects that make PHAs intrinsically natural and green materials in all stages of their life cycle. The authors show that PHAs are bio-based, biosynthesized, biocompatible, and biodegradable in all relevant environmental settings, and they are compostable both at home and in industry. It is demonstrated that PHAs are an ideal replacement for fossil plastics, as they provide us with the benefits of conventional plastics and meet all the necessities of a truly circular bioeconomy. It is elaborated how the production, consumption, and end-of-life scenarios of PHAs are embedded in the well-known 12 Principles of Green Chemistry, which constitute the basis for the sustainable manufacturing of polymers needed by humankind [77].

Several articles in the Special Issue deal with PHA production from CO_2_ and methane as abundant C1 carbon sources. In this context, Rogerio Ramos de Sousa Junior, Fabiano Eduardo Marques Cezario, Leonardo Dalseno Antonino, and Demetrio Jackson dos Santos from Santo André, Brazil, in collaboration with Maximilian Lackner from Vienna, Austria, compared two samples of P(3HB) synthesized through two different autotrophic routes. The first P(3HB) sample was produced photoautotrophically by the cyanobacterium *Synechocystis* sp. PCC 6714; here, CO_2_ was used as carbon source, while light delivered the needed energy. In contrast, the second biopolyester was accumulated chemoautotrophically by the methanotrophic bacterium *Methylocystis* sp. GB 25 by using methane as carbon and energy source. Material characterization of both biopolyesters showed that the products displayed similar thermal and mechanical properties as reported for standard P(3HB) samples from heterotrophic cultivations. Importantly, authors emphasize that both gaseous substrates, CO_2_ and methane, when used as substrates for PHA production, are highly attractive due to the process scalability, the widespread availability of these gases, and their favorable growth rates, especially in the case of methanotroph. However, both processes require specialized bioreactors for gas fermentations, with cyanobacteria requiring photobioreactors and methanotrophs necessitating continuous-flow bioreactor systems. Hence, further optimization of bioreactors and cultivation regimes is indispensable to minimize substrate losses and reduce the overall PHA production costs [78].

Chih-Ting Wang, Ramamoorthi M Sivashankari, and Yuki Miyahara from the laboratories of Takeharu Tsuge in Yokohama, Japan, used recombinants of the well-described facultatively chemoautotrophic bacterial strain *Cupriavidus necator* H16, an organism utilizing both CO_2_ and the heterotrophic carbon source fructose, but not glucose. Starting from a PHA-negative mutant, authors designed a leucine analog-resistant strain (1F2), which, surprisingly, was able to synthesize 3-hydroxybutyrate (3HB)-based PHA copolyesters containing 3-hydroxyvalerate (3HV) and branched 3-hydroxy-4-methyvalerate (3H4MV) monomeric building blocks both autotrophically (from CO_2_) or heterotrophically (fructose). This is explained by 1F2 expressing a broad substrate-specific PHA synthase and tolerating feedback inhibition of branched amino acids. To increase incorporation of building blocks different to 3HB, 1F2 was subjected towards further genetic engineering, addressing several modifications, such as overexpression of genes encoding the key enzymes 3-ketothiolase, 2-ketoacid decarboxylase, and phenylacetaldehyde dehydrogenase, which resulted in increased 3HV formation. Additional deletion of the 3-hydroxyacyl-CoA dehydrogenase gene (*hbdH*) enabled production of increased production of the monomer 3-hydroxy-2-methylpropionate (3H2MP), and remarkably increased PHA molecular mass when utilizing fructose [79].

Kenji Tanaka, Izumi Orita, and Toshiaki Fukui, also based in Japan, contribute a study using recombinant *C. necator* for efficient and high-titer production of P(3HB-*co*-3HHx), a material currently produced at large industrial scale from vegetable oils, but not yet produced from inexpensive carbon sources. This PHA copolyester is commercially among the top selling representatives of the “PHAmily”, as it is flexible, soft, has a wide “processing window” (temperature difference between melting point and decomposition temperature), and is readily marine-biodegradable. In present study, in contrast to previous works carried out exclusively on shaking flask scale, highly efficient P(3HB-*co*-3HHx) production from CO_2_ as the sole carbon source (used substrate gas mixture: H_2_/O_2_/CO_2_ = 80:10:10 *v*/*v*%) was studied by pH-stat cultivation of recombinant *C. necator* in a recycled-gas, closed-circuit bioreactor system. Phosphate- and nitrogen-limited cultivation conditions to boost PHA biosynthesis were studied, both yielding high P(3HB-*co*-3HHx) concentrations of about 50 to 60 g/L after 216 h of cultivation. The authors emphasize that this study might pave the way towards mass production of P(3HB-*co*-3HHx) from problematic, industry-derived CO_2_ [80].

A follow-up study by the same group of experts achieved exceptionally high P(3HB-*co*-3HHx) concentration of about 85 g/L with recombinant *C. necator* after a considerably shorter cultivation time of only 148 h. A bioreactor equipped with a basket-shaped agitation device was used to enhance oxygen transfer in the culture medium; gases with higher O_2_ portions were continuously supplied in order to maintain the gas composition constant. The concentrations of ammonium (nitrogen source) and phosphate in the culture medium were maintained at low levels to provoke PHA biosynthesis. The obtained 3HHx monomer contents in (3HB-*co*-3HHx) copolyesters were 10–11 mol-%, matching the needs of practical applications [81].

The co-production of two marketable bioproducts, namely gluconic acid, a compound of food technological and pharmaceutical significance, and P(3HB), was investigated by Tânia Leandro, M. Conceição Oliveira, M. Manuela R. da Fonseca, and M. Teresa Cesário from Lisboa, Portugal. The moderately halophilic bacterium *Halomonas elongata* 1H9^T^ was cultivated in fed-batch mode using glucose as sole carbon source, while nitrogen limitation was chosen as factor supporting the shift of metabolites towards PHA biosynthesis. Under these conditions, a maximum P(3HB) content in biomass of 53 wt.-% and a maximum gluconic acid titer of 133 g/L of were obtained. In contrast, phosphate limitation resulted in only modest P(3HB) accumulation, while no production of gluconic acid was observed at all. The authors highlight that the co-production of intracellular P(3HB) and extracellular gluconic acid, a product of high commercial significance, might be a decisive step towards cost-efficient PHA production [82].

Another halophilic organism used for PHA production is the extremely salt-requiring haloarchaeon *Haloferax mediterranei*. This prototype archaeal PHA producer constitutes a real halophilic cell factory, as it not only synthesizes PHA copolyesters, but also other marketable bioproducts such as pigments (bacterioruberin), antibacterial halocins, and xanthan-like polysaccharides [83]. The Spanish research team encompassing Lorena Simó-Cabrera, Salvador García-Chumillas, Sergio J. Benitez-Benitez, Verónica Cánovas, Fuensanta Monzó, Carmen Pire, and Rosa María Martínez-Espinosa used this strain for cellular growth and P(3HB-*co*-3HV) bioproduction from organic leftovers from the candy industry (waste materials which are still underexplored as a biotechnological feedstock). P(3HB-*co*-3HV) concentrations of 0.26 and 0.98 g/L were obtained when using two different qualities of candy industry waste differing in sugar, protein, and salt content in shaking flask experiments, demonstrating for the first time the possibility to valorize candy waste for biopolyester production [84].

Finally, an analytical article was contributed by Kyo Saito, M. Venkateswar Reddy, Omprakash Sarkar, A. Naresh Kumar, DuBok Choi, and Young-Cheol Chang, an authorship spread between the Republic of Korea, the US, Sweden, and Japan. This work deals with a novel method to rapidly quantify the monomeric compositions of the copolyester P(3HB-*co*-3HV) produced by *Bacillus* sp. CYR1 from acetate and valerate, and the homopolyester P(3HV) produced by the bacterium *Chromobacterium violaceum* using valerate as sole carbon source. The obtained polyesters were subjected towards alkaline hydrolysis, and hydrolysis products were determined using High-Performance Liquid Chromatography (HPLC). It was shown that alkaline decomposition of P(3HB-*co*-3HV) generates the unsaturated compounds 2-butenoic acid and 2-pentenoic acid, which can conveniently be quantified via HPLC [85]. Importantly, the HPLC results obtained by the new method were comparable with results obtained using the well-established gas chromatography (GC-FID) analysis, which goes back to the 1970s, and currently is applied as the global gold standard for PHA determination in innumerable laboratories, but is more time- and labor-intensive by far [86].

## 3. Conclusions

As take-home message, the contributions to this Special Issue, “Advances in Polyhydroxyalkanoate (PHA) Production, Volume 4”, address highly topical research and development activities in the field of intrinsically natural PHA biopolyesters of diverse composition and microbial origin. It is especially shown that PHA production from abundant gaseous substrates is on an auspicious way to catch up with established heterotrophic cultivation regimes in terms of productivity and product quality. Here, contributions clearly illustrate that optimized bioengineering, especially bioreactor design and operation mode, are the *conditio sine qua non* to lift these autotrophic processes to the level of industrial maturity. It is also shown that tools of genetic engineering and synthetic biology are rapidly gaining importance for production of PHAs of tailor-made composition from carbon substrates as simple and abundant as possible, such as CO_2_ from industrial effluent gas, methane from anaerobic digestion of organic waste materials, and syngas (easily accessible from diverse organic rejects). We also learn that efficient extremophilic wild-type strains, both bacteria and haloarchaea can be chosen as workhorses in the production of PHAs and marketable side-products from inexpensive raw materials, even in open and unsterile cultivation setups, thus contributing to biorefinery concepts. Moreover, novel analytical tools presented here will pave the way towards rapid process control in the context of fine-tuning the monomeric composition of PHAs, which in turn is decisive to produce biopolyesters of pre-defined properties.

As Guest and Section Editor, I am highly confident that present fourth PHA-related Special Issue of *Bioengineering* will again encourage researchers around the world to carry out and intensify further research and development activities in the field of these fascinating biopolyesters.
